# Urban Traumatic Moving Injuries Before and During SARS-CoV-2: A Multilinear Regression Analysis

**DOI:** 10.7759/cureus.36905

**Published:** 2023-03-30

**Authors:** Geena Jung, Vincent Giordano, Tara Harrington, Lance Jung

**Affiliations:** 1 Trauma Surgery, Albert Einstein College of Medicine, New York, USA; 2 Trauma Services, Richmond University Medical Center, New York, USA; 3 Trauma Surgery, Richmond University Medical Center, New York, USA

**Keywords:** injury mechanism, length of hospital stay (los), ambulance transport, covid 19, general trauma surgery

## Abstract

Background

The onset of the coronavirus pandemic (COVID-19/SARS-CoV-2) saw an overall decline in traffic. Fundamental shifts in the pattern of traffic-related traumas were observed across the United States and beyond.

Objectives

This study aims to predict changes in the length of stay (LOS) for patients sustaining traumatic moving injuries before and during the coronavirus pandemic.

Methods

All moving injuries (bicycle accidents, pedestrians struck, motor vehicle/motorcycle accidents) before and during the first SARS-CoV-2 wave in the US were extracted from our hospital’s trauma registry. The study period was from March 1st to October 31st of 2019 and 2020, respectively. Ordinary least squares (OLS) multilinear regression models were estimated with a significance level of 0.05.* *

Results

In both periods, the Glasgow coma scores (GCS), ICU LOS, injury severity scores (ISS), and admitting service had significant impacts on hospital duration. Higher GCS scores increased the hospital LOS by 0.811 days in 2019 and 0.587 days in 2020. A higher ISS resulted in an increase in LOS by 0.207 days in 2019 and 0.124 days in 2020. The ICU admissions increased LOS by 0.82 days in 2019 and 1.25 days in 2020. Admissions to trauma services increased in duration by 2.111 days in 2019 and 1.379 days in 2020. Average LOS dropped from 3.09 to 2.50 days between both periods.

Conclusion

Our trauma center saw significant changes in the admission patterns of moving injuries during COVID-19. We must therefore be better prepared to handle increased volume during public health emergencies and potential reductions in trauma utilization. Local injury prevention efforts may help reduce the burden on trauma centers during such emergencies as they did during COVID-19, allowing for greater focus on non-trauma patients.

## Introduction

The onset of the coronavirus pandemic (SARS-CoV-2) in late 2019 into early 2020 impacted the provision of core medical services in the United States and beyond. Hospitals and clinics were initially overwhelmed by the surge of infections. An intriguing byproduct of resulting public health measures was reduced vehicle traffic. In the first 30 days of the pandemic’s major onset in the United States, the Bureau of Transportation Statistics reported that the passenger vehicle miles traveled (VMT) index dropped from a baseline level of 1.0 on March 15th, 2020 to a low of 0.4 by April 12th, 2020 [1].

In many jurisdictions both domestic and beyond, this reduced traffic volume resulted in an overall decline in reported traumatic motor vehicle accidents (MVAs). Health authorities in Ontario, Canada reported that MVA traumas for patients aged 80 and older declined by 64.7%, while the same injuries for those aged 35 to 54 years declined by 22.9%, though public health countermeasures appeared to have had a negligible impact on MVA traumas for age groups below 35 and between 55 to 79 [2]. In contrast, research conducted in Connecticut on the eight-week “stay-at-home” order implemented by the governor’s office documented an increase of 28 additional MVAs relative to preceding years [3]. A review of statewide data in Alabama, USA reported a similar increase in MVAs, with speeding and intoxicated vehicle operation constituting the largest share of these traumas [4]. 

Generally, patients suffering traumatic injuries often face longer inpatient hospital stays than those with non-traumatic characteristics such as planned operations or strokes/seizures. Several factors affect hospital length of stay (LOS) for trauma patients. For instance, some research indicates that among geriatric patients, placement issues at skilled nursing facilities (SNFs) skewed the average LOS for trauma patients higher [5]. Other studies have pointed to the role played by the type of traumatic injury sustained and insurance coverage in hospital LOS. According to a study done on trauma admissions between 2006 and 2010 at a level 1 center, blunt injuries, those with Medicaid/Medicare or self-paying, and older patients had excessively longer hospital durations than their counterparts [6]. The relationship between Medicaid coverage and LOS is disputed by Holzmacher et al., who found that expanded Medicaid enrollment was associated with an average decrease in LOS of about 3.32 days [7]. Interestingly, some research indicates that seemingly obvious factors such as injury severity do not have a statistically significant relationship to hospital duration [8], while a level 1 trauma center in New Jersey saw a 37.2% fall in ISSs that coincided with a 59.7% reduction in total LOS in hours [9].

However, medical institutions saw noticeable changes in their general trauma admissions during the pandemic. Hospitals in Los Angeles County, California saw an increase in admissions for penetrating traumas while a decrease in blunt traumas was noted. Generally, however, there was an overall decrease in hospitalizations for traumatic injuries from March 1st, 2020 to June 7th, 2020. A 55% reduction in traffic is partially responsible for the fall in blunt traumas [10]. The average LOS also decreased by approximately 3.6% across 18 states, with noted increases in penetrating traumas and burns [11]. A study of two level 1 trauma centers in Santa Clara County, California reported that trauma activations were nearly five times higher in 2018 and 2019 than during the governor's stay-at-home order initiated on March 16^th^, 2020 [12]. Only one out of the 81 activations during the stay-at-home order tested positive for COVID-19.

Data from a New Zealand level 1 trauma center demonstrates a similar downward adjustment in trauma admissions. Overall, this trauma center saw a 43% average fall in trauma admissions during the level 4 lockdowns in April 2020. The center also saw major traumas halve over this 14-day lockdown period, though traumatic falls in nursing facilities and at home remained prevalent [13]. 

While there was an overall decrease in hospitalizations for trauma-related injuries, more aggressive and high-energy injuries were reported throughout the pandemic. In particular, data from a level 1 trauma center in New York showed a 117% increase in gunshot wounds (GSWs), a 110% increase in motorcycle crashes, and a 108% increase in strikes by blunt objects from March 1st, 2020 to September 1st, 2020 [14]. Interestingly, there was a 58% decrease in the number of ICU patients, which was believed to be a direct result of decreased bed space to accommodate COVID-19 patients. Research on motor vehicle collisions during the initial country-wide lockdown in Japan in March 2020 shows increased aggressive driving which triggered more fatalities [15]. Reporting by Reuters supports the conclusions of the previous New York and Japan studies, which showed more aggressive traumas being reported during the initial lockdowns coupled with more careless driving [16]. 

In this paper, we sought to analyze the effects the SARS-CoV-2 pandemic had on traumatic moving injuries (bicycle accidents, pedestrians struck, motor vehicle accidents, and motorcycle accidents) and associated hospital LOS in a level 1 trauma center. Data on all moving injuries before and during the early wave of the pandemic from March 1st to October 31st of 2019 and 2020, respectively, were extracted from the Richmond University Medical Center’s trauma registry. Two separate ordinary least squares (OLS) multilinear regression models of LOS were then created and estimated with a significance level of $\alpha = 0.05$. 

## Materials and methods

This retrospective study was performed on data collected at the Richmond University Medical Center in New York, USA. The protocol for this paper (approval no. 19171) was reviewed and approved by New York Medical College’s Institutional Review Board under exempt review. The methods of data collection complied with patient and hospital privacy laws. 

This paper intends to predict hospital LOS. Specifically, it is to predict LOS for traumatic moving injuries before and during the initial major SARS-CoV-2 outbreak in the United States in March 2020. Our study classifies moving injuries into four classes: 1) pedal bicycle riders/skateboarders injured in any accident; 2) scooter/electric scooter riders injured in any accident; 3) pedestrians struck by vehicles, scooters, trucks, buses, or any other mobilized transportation; and 4) motorcycle/moped riders, car drivers/passengers injured in any accident. Other classes, such as bus accidents, tractor-trailer accidents, etc. were grouped into the fourth category. To predict LOS both before and during the early wave of the pandemic, two separate OLS multilinear regression models were developed. A total of eight variables were included in both models to produce a base regression formulation as seen in the following equation: 

$\lambda_t = \omega_0 + \omega_1x_1 + \omega_2x_2 + ... + \omega_8x_8 + \varepsilon_0$

In the equation, $\lambda_t$ is hospital LOS in days at year $t$, $x_1 ... x_8$ are the predictor parameters used in the model, $\omega_0$ is the constant term, or the equation value if all parameters were equal to zero, $\omega_1 ... \omega_8$ are the coefficients on the predictor variables, and $\varepsilon_0$ is the disturbance term or the measure of variance between the model and the predicted population. 

The initial model contained eight exogenous variables as seen in Table 1. 

**Table 1:** Theoretical estimators for both models GCS: Glasgow coma scale/core; ICD-10: International Classification of Diseases, tenth revision; ISS: Injury severity score; LOS: Length of stay; EMS: Emergency medical services

Variable	Description	Predicted impact on LOS
x_1_	GCS upon arrival	Negative (-)
x_2_	Patient's age at the time of the visit	Positive (+)
x_3_	ICD-10 ISSs	Unknown (+/-)
x_4_	LOS in the ICU	Positive (+)
x_5_	Biological sex (binary variable): 1 = Male, 0 = Female	Unknown (+/-)
x_6_	EMS transport: 1 = EMS, 0 = Walk-In	Positive (+)
x_7_	Admitting service: 1 = Trauma, 0 = Non-trauma	Positive (+)
x_8_	Insurance type: 1 = Medicare/Medicaid, 0 = All others	Unknown (+/-)

The variables for age, ICU LOS, EMS transportation, and trauma services admission are expected to track strongly with $\lambda_t$. Older patients tend to have more preexisting conditions and hospital complications, while ICU LOS is directly tethered to overall LOS. Those ambulating on their own to the ER tend to have less severe injuries than patients requiring EMS transport, potentially reducing average LOS. Typically, those with more severe injuries are admitted to trauma services while those needing basic observations tend to be admitted to internal medicine. On the other hand, more alert patients score higher on the GCS and typically require fewer interventions or stays. However, ISSs could push the model in either direction, as some severe injuries produce low ISSs while correlating with more hospital complications (e.g., GSWs with post-surgery complications), while other injuries with higher ISSs may warrant fracture reductions that require recovery/step-down room monitoring (e.g., long bone fractures). It is also unclear if insurance will correlate with $\lambda_t$, though patients with private insurance may face fewer discharge obstacles, especially regarding skilled nursing placements.

Raw data was confidentially queried and randomized by the department of trauma services. Data was collected on all moving injuries trauma patients admitted, discharged, expired, and who left against medical advice from March 1^st^, 2019 to October 31^st^, 2019, and then again from March 1^st^, 2020 to October 31^st^, 2020. The following raw data was initially collected: 1) arrival date; 2) hospital duration in total days; 3) emergency department-recorded GCS; 4) patient age at the time of visit; 5) ISS; 6) LOS in the ICU; 7) biological sex; 8) mode of transportation; 9) admitting hospital service; and 10) primary insurance coverage. Total observations in this study were $i_{2019} = 177$ and $i_{2020} = 146$, an approximately 21% decrease.

The model contained four nominal variables (mode of transport, sex, admitting service, and insurance), which were assigned new binary classes i.e., for the mode of transport: 1 = EMS and 0 = walk-ins; for sex: 1 = male and 0 = female; for insurance: 1 = Medicare/Medicaid and 0 = all others; for admitting service: 1 = trauma services and 0 = all other services. Registry numbers, arrival dates, and any other identifying patient information were removed to ensure patient privacy and Health Insurance Portability and Accountability Act (HIPAA) compliance.

An a priori power analysis was carried out using G*Power software to determine the minimum sample size for both study periods. Using a fixed-model F-test with an estimated effect size of $f^2 = 0.15$, a power level of 80%, and $\alpha = 0.05$, a minimum sample size of $i_t = 114$​ would be sufficient to conduct an analysis of ample power. All data, models, and charts were analyzed using Stata/BE 17 statistical software (StataCorp LLC, College Station, TX, USA).

## Results

Core statistical tests were performed to ensure the model's goodness of fit. A Breusch-Pagan/Cook-Weisberg test was used to detect the presence of heteroskedasticity. For 2019, the test produced a test statistic $\chi^2 = 50.24$ and a resulting p-value of 0.001, well below the significance threshold $\alpha = 0.05$. For 2020, $\chi^2 = 19.64$ and a germane p-value of 0.001 were returned, indicating non-constant variance of the residuals. Thus, heteroskedasticity was detected in both years. To correct this, robust standard errors were incorporated, and the model was run again. Variance inflation factors (VIFs) were calculated with a threshold of 5 established as the critical value. In 2019, the variable for ISSs had the highest factor of 2.43, and the binary variable for sex returned the lowest at 1.05, producing a mean VIF of 1.40. For 2020, extrema were calculated at 3.03 for ISS and 1.05 for the EMS transportation variable respectively, with a mean of 1.56. Thus, no collinearity was detected in either period and no variables were initially excluded.

For 2019, the model produced an initial $R^2 = 0.5639$. At the $\alpha = 0.05$ significance level, the variables age, sex, EMS transport, and public insurance were not statistically significant. After eliminating these variables, the following results were obtained as seen in Table 2.

**Table 2:** Final regression results for 2019 R^2^: Coefficient of determination (the proportion of the length of stay predicted by the model), GCS: Glasgow coma scale/score, ISS: Injury severity score, LOS: Length of stay, p-value: Probability value, CI: Confidence interval

R^2^	0.5479				
Observations	177				
Variable	Coefficient	Robust errors	p-value	95% CI	95% CI
GCS	0.811	0.349	0.021	0.122	1.499
ISS	0.207	0.072	0.004	0.066	0.348
ICU LOS	0.816	0.124	0.001	0.572	1.059
Trauma admission	2.111	0.492	0.001	1.141	3.082
Constant	-11.678	5.362	0.031	-22.263	-1.094

This resulted in a multiple linear regression model computed as follows:

$\lambda_{2019} = -11.678 + 0.811x_1 + 0.207x_3 + 0.816x_4 + 2.111x_7$

For 2020, the intention was to isolate the impact of coronavirus on the model. The regression was run with the same variables as in 2019, producing an initial $R^2 = 0.6677$. Using a 95% confidence interval (CI), the same variables as in 2019 were not significant in 2020: age, sex, EMS transportation, and public insurance. Table 3 displays the final output after excluding the non-significant parameters. 

**Table 3:** Final regression results for 2020. R2: Coefficient of determination (the proportion of the length of stay predicted by the model), GCS: Glasgow coma scale/score, ISS: Injury severity score, LOS: Length of stay, p-value: Probability value, CI: Confidence interval

R^2^	0.6440				
Observations	146				
Variable	Coefficient	Robust Errors	p-value	95% CI	95% CI
GCS	0.587	0.214	0.007	0.164	1.009
ISS	0.124	0.047	0.009	0.031	0.217
ICU LOS	1.254	0.053	0.001	1.149	1.358
Trauma admission	1.379	0.482	0.005	0.425	2.332
Constant	-7.853	3.277	0.018	-14.331	-1.375

This produced a linear regression equation as follows: 

$\lambda_{2020} = -7.853 + 0.587x_1 + 0.124x_3 + 1.254x_4 + 1.379x_7$

Finally, the following core averages and changes from 2019 to 2020 were observed in Table 4.

**Table 4:** Descriptive statistics changes between 2019 and 2020 $\lambda_t$: Total hospital duration at time *t*, ISS: Injury severity score, %∆: Percentage change

Tabulation	2019	2020	%∆
Average λ_t_	3.09	2.50	-19.09%
Average ISS	6.03	6.02	-0.17%
Average age	41.19	37.64	-8.62%

**Figure 1:**
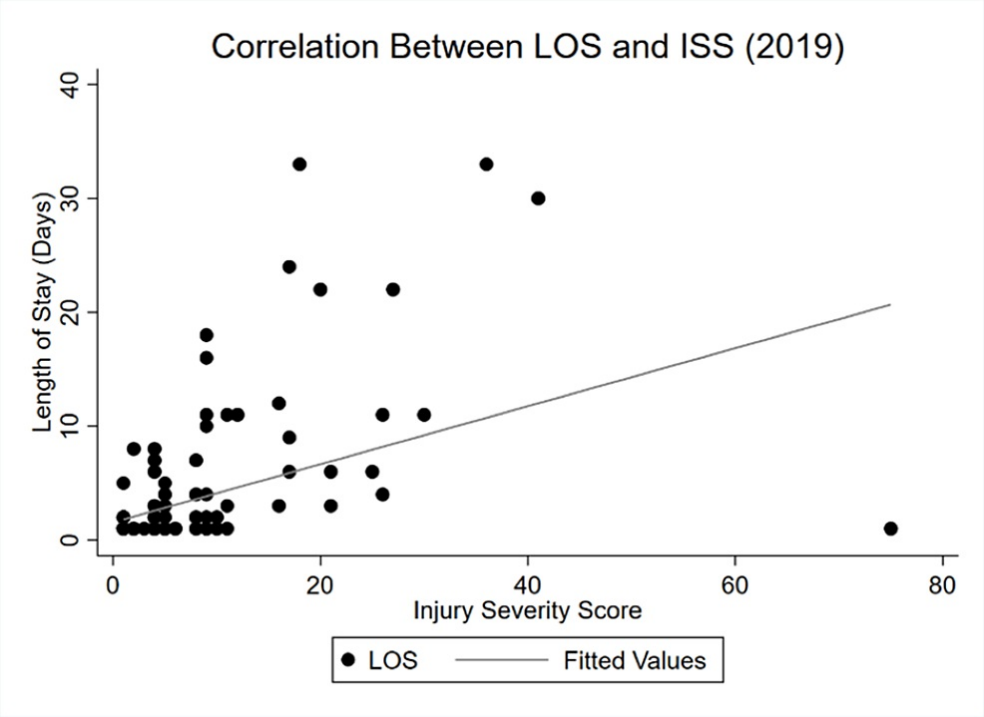
LOS and ISS correlation in 2019 ISS: Injury severity score, LOS: Length of stay

**Figure 2:**
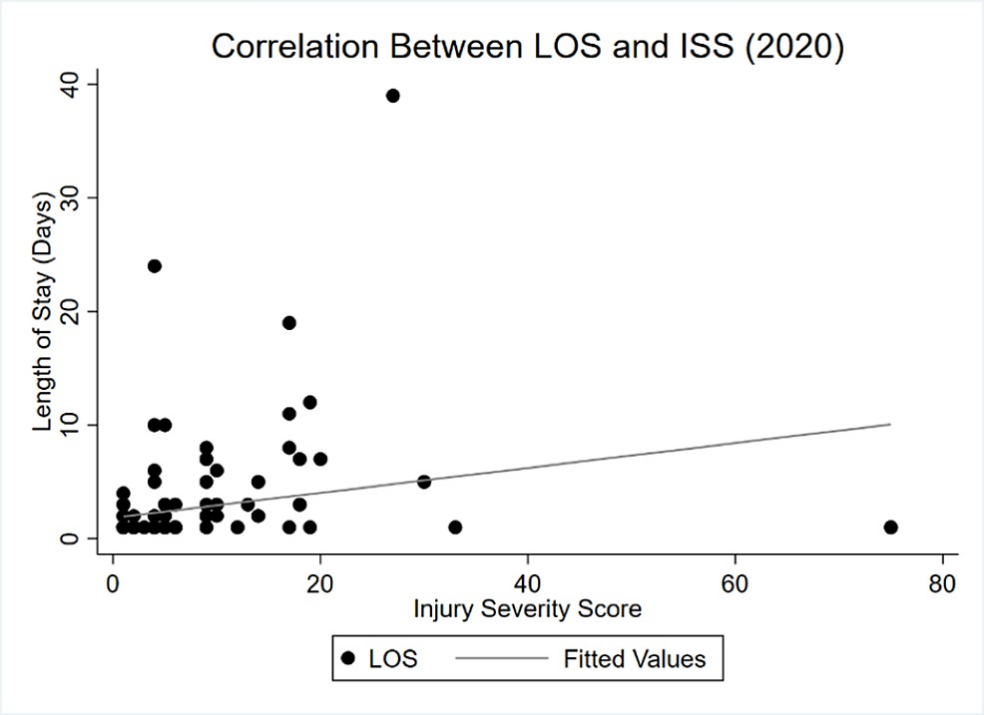
LOS and ISS correlation in 2020 ISS: Injury severity score, LOS: Length of stay

Correlations between $\lambda_t$ and the significant continuous variables of GCS, ICU LOS, and ISS were analyzed to compare the magnitude sizes in both years, resulting in more inelastic values for GCS and ISS and a negligibly more elastic curve for ICU LOS. Figure 1 and Figure 2 display the changes in correlations between 2019 and 2020.

**Figure 3:**
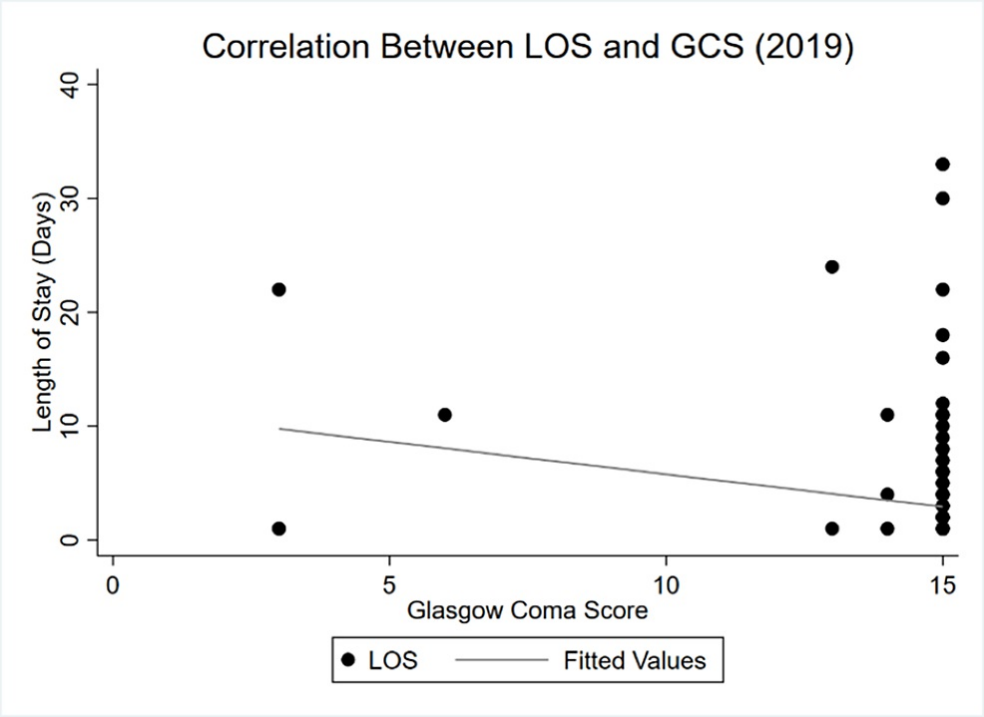
LOS and GCS correlation in 2019 GCS: Glasgow coma scale/score, LOS: Length of stay

**Figure 4:**
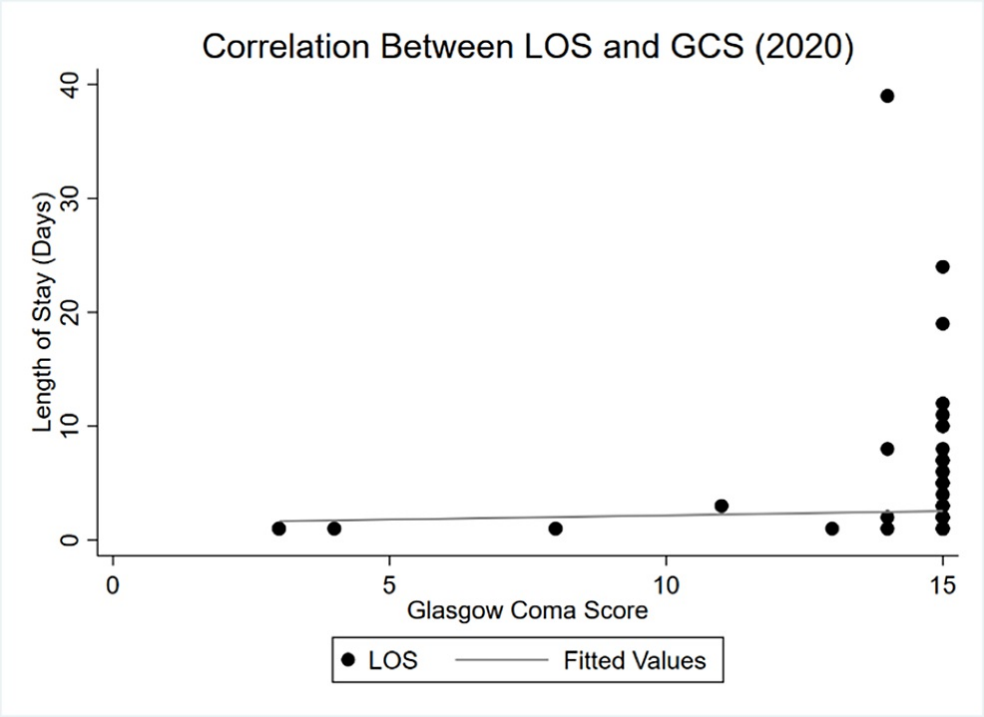
LOS and GCS correlation in 2020 GCS: Glasgow coma scale/score, LOS: Length of stay

The correlation between LOS and injury severity weakened during COVID-19. While both slopes demonstrate a positive dependency of LOS on ISS, lockdowns seemed to have made the curve more inelastic. Changes in injury severity had a proportionally smaller impact on overall LOS in 2020 relative to 2019. Figure 3 and Figure 4 show the shifts between LOS and GCS between both periods. 

**Figure 5:**
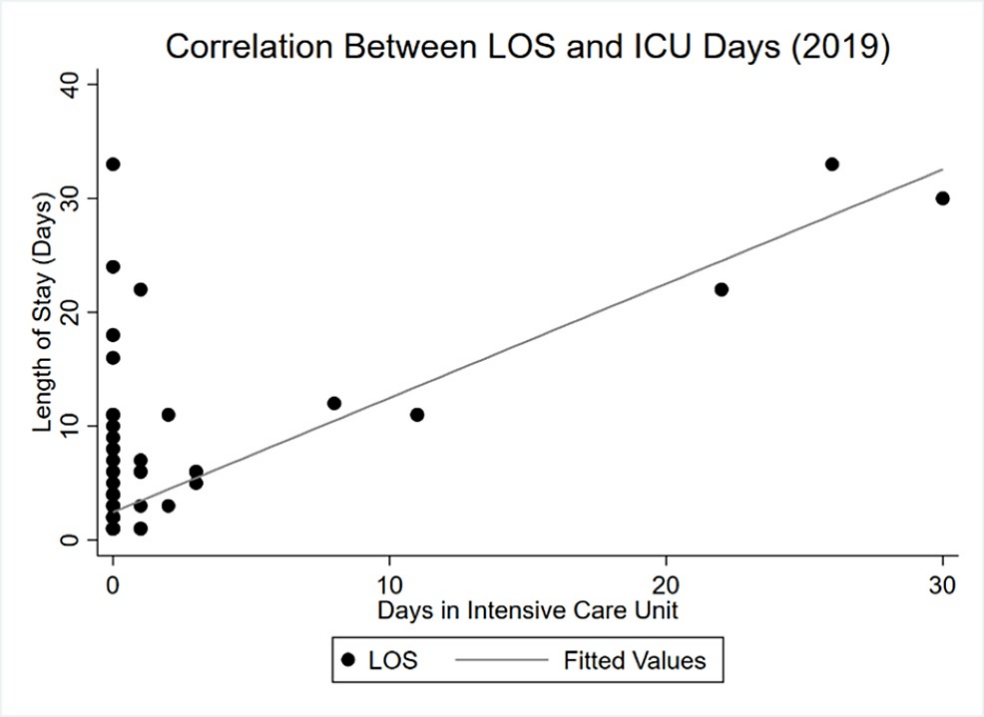
LOS and ICU LOS correlation in 2019 LOS: Length of stay

**Figure 6:**
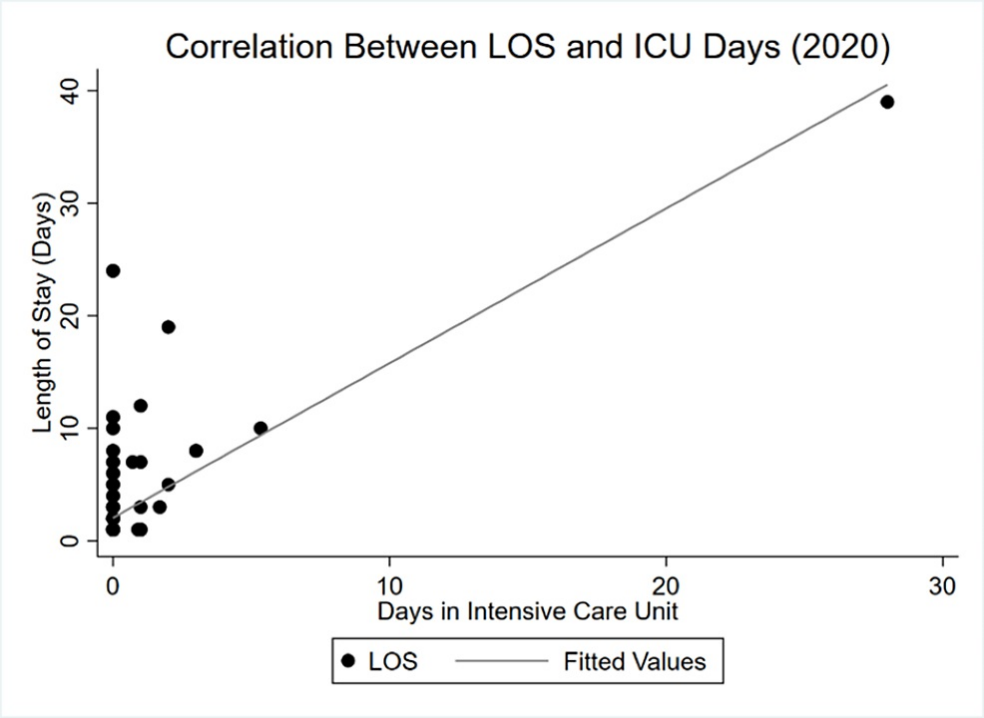
LOS and ICU LOS correlation in 2020 LOS: Length of stay

By 2020, the correlation between LOS and GCS became almost perfectly inelastic. Whereas there was a negative, albeit highly inelastic relationship in 2019, an even more inelastic yet positive dependency is observed during COVID-19. Finally, Figure 5 and Figure 6 show the shifts between LOS and ICU LOS.

The relationship between the overall LOS and days in the ICU remained relatively stable compared with the preceding variables. Yet the decrease in average LOS documented in the results section is illustrated by the reduced spread in observed values from Figure 5 to those in Figure 6. More values cluster below 10 days in 2020 than they do in 2019.

## Discussion

By generating two separate regression models of hospital LOS for 2019 and 2020, we sought to predict the effects that the SARS-CoV-2 pandemic had on traumatic moving injuries and associated LOS in a level 1 trauma center. For most of the statistically significant variables in both 2019 and 2020 (ISS, ICU LOS, and trauma aervices admission), the results were consistent with what had been predicted. Older patients tend to have more preexisting conditions and hospital complications, contributing to a longer LOS. Moreover, those ambulating on their own to the emergency department tend to have less severe injuries than patients requiring EMS transport, potentially reducing average LOS. Finally, those with more severe injuries are typically admitted to trauma services while those needing basic observations tend to be admitted to non-surgical services such as internal medicine and pediatrics. 

The impact of ED-recorded GCS on hospital LOS was surprising. It was expected that patients with lower GCSs would require more treatment than more alert and oriented patients. Yet GCS had a positive impact on total hospital duration. One potential explanation is the range of GCS values having a significant impact. Approximately three-fourths of patients had a GCS of either 15 or 14, signifying a heavy skew towards more alert patients. Thus, the sheer magnitude of alert and oriented patients weakened the effect of lower GCSs. Additionally, the decrease in coefficient magnitude between both periods may be attributable to increased discharges of high-GCS patients to make room for COVID-19 patients. 

The variable with the most impact on hospital LOS in both 2019 and 2020 was the admitting service/department. In 2019, admission to trauma services resulted in an additional 2.1 days in the hospital compared to other services such as orthopedics and neurosurgery. Interestingly, admitting service played less of a role in 2020, with an admission to trauma services resulting in only 1.5 additional days in the hospital, a 37.5% drop relative to 2019. The decrease in the impact admitting service had on LOS may be explained by the overwhelming patient volume during the initial pandemic wave, which resulted in more frequent and quicker discharges of trauma patients to make room for quarantine units. The increase in ICU LOS magnitude due to extended respiratory observations supports this conclusion.

There was a negligible decrease in average injury severity of -0.17%. It was predicted that urban public health controls would have reduced vehicle traffic, resulting in fewer traumatic moving injuries and, thus, less severe injuries overall. However, it appears that while injury mechanisms changed during COVID-19, the particular injuries sustained remained static, resulting in the trivial ISS change.

This study demonstrated that irrespective of public health emergencies, individuals will continue to sustain traumatic moving injuries. Therefore, certain targeted prevention programs would prove beneficial in the long run. Our trauma center's injury prevention (IP) program conducts regular car seat checks for interested community members. This ensures local and state traffic law compliance while reducing moving injury frequency and the potential for traumas among children. Expanding this program to other jurisdictions is a long-term objective of our trauma center. Another IP effort is the American College of Surgeons' Stop the Bleed program, of which our trauma center is a member. This program teaches community members and leaders how to reduce blood loss during traumas, and an expansion of this program would prove beneficial to reducing injury and mortality from high-energy moving traumas.

Overall, the onset of the SARS-CoV-2 pandemic has had a dramatic effect on the traumatic injuries faced by those in the US, and in this study, we show its effects on moving injuries and associated LOS in the hospital. While this study is entirely retrospective in its data collection, it is nevertheless predictive in its analysis. As urban population densities in New York and other regions increase, medical institutions and policymakers alike must be vigilant in identifying what key elements may potentially overwhelm healthcare providers, especially during public health emergencies By doing so, they may reasonably decrease the potential future stresses placed on providers and their networks. 

Limitations

We acknowledge that there are several limitations to our study. The use of OLS regression was predicted as the best estimation method for the analysis carried out in this paper. However, other estimations such as non-linear models may have proven more useful given the detection of heteroskedastic residuals early on. Additionally, incomplete registry data from 2019 hampered the inclusion of other variables that may have been analytically useful. For example, a category parameter for injury zip codes may have helped identify which areas see more moving traumas. However, trauma services reported that zip codes from either EMS or patients were unavailable or not recorded for many patients. Another potential parameter is injury diagnosis. Several variables could have been created from International Classification of Diseases, tenth revision (ICD-10) codes for traumatic injuries, such as binary indicators for cranial traumas or categorical indicators. Yet given the trivial shift in ISS during COVID-19, including this variable may have been redundant. Nevertheless, despite the potential limitations of the model, great care was taken to clean the data and produce a cohesive regression for both 2019 and 2020.

Another limitation is the acute nature of the study. The research was conducted at a single level 1 trauma center. However, more studies should be replicated at neighboring institutions in other New York City boroughs and beyond to test whether the changes seen at our trauma center hold elsewhere. Future research should also seek to investigate what impacts augmented injury prevention efforts have on reducing urban traumatic moving injuries. 

## Conclusions

The impact of SARS-CoV-2 has been felt acutely across the US healthcare provider network. Nowhere has this impact been larger than in urban cores such as New York City and Los Angeles. The beginning of the coronavirus pandemic in March 2020 resulted in major changes to the frequency and underlying characteristics of traumatic moving injuries. Through a rigorous statistical analysis of moving injury traumas on Staten Island before and during the SARS-CoV-2 initial outbreak, core shifts in injury characteristics, hospital duration, and demographics were observed. Healthcare providers at trauma centers nationwide should consider the impact of the scope and type of traumatic moving injuries they treat when determining the proper LOS for the patients in question.
